# Bacterial and Archaeal Communities in Erhai Lake Sediments: Abundance and Metabolic Insight into a Plateau Lake at the Edge of Eutrophication

**DOI:** 10.3390/microorganisms12081617

**Published:** 2024-08-08

**Authors:** Zhen Xie, Wei Li, Kaiwen Yang, Xinze Wang, Shunzi Xiong, Xiaojun Zhang

**Affiliations:** 1 State Key Laboratory of Microbial Metabolism, and Joint International Research Laboratory of Metabolic & Developmental Sciences, School of Life Sciences and Biotechnology, Shanghai Jiao Tong University, Shanghai 200240, China; xiezhen07@sjtu.edu.cn (Z.X.); kevinyang97@outlook.com (K.Y.); 2 National Observation and Research Station of Erhai Lake Ecosystem in Yunnan, Dali 671000, China; andynee@foxmail.com (W.L.); xinzewang@sjtu.edu.cn (X.W.); xiongshunzi@sjtu.edu.cn (S.X.); 3 Yunnan Dali Research Institute, Shanghai Jiao Tong University, Dali 671000, China

**Keywords:** bacterial and archaeal communities, rare and abundant taxa, metabolic potential, eutrophication indicators, mesotrophic plateau lake

## Abstract

The littoral zones of lakes are potential hotspots for local algal blooms and biogeochemical cycles; however, the microbial communities within the littoral sediments of eutrophic plateau lakes remain poorly understood. Here, we investigated the taxonomic composition, co-occurrence networks, and potential functional roles of both abundant and rare taxa within bacterial and archaeal communities, as well as physicochemical parameters, in littoral sediments from Erhai Lake, a mesotrophic lake transitioning towards eutrophy located in the Yunnan–Guizhou Plateau. 16S rRNA gene sequencing revealed that bacterial communities were dominated by Proteobacteria, Bacteroidetes, and Chloroflexi, while Euryarchaeota was the main archaeal phylum. Co-occurrence network analysis revealed that keystone taxa mainly belonged to rare species in the bacterial domain, but in the archaeal domain, over half of keystone taxa were abundant species, demonstrating their fundamental roles in network persistence. The rare bacterial taxa contributed substantially to the overall abundance (81.52%), whereas a smaller subset of abundant archaeal taxa accounted for up to 82.70% of the overall abundance. Functional predictions highlighted a divergence in metabolic potentials, with abundant bacterial sub-communities enriched in pathways for nitrogen cycling, sulfur cycling, and chlorate reduction, while rare bacterial sub-communities were linked to carbon cycling processes such as methanotrophy. Abundant archaeal sub-communities exhibited a high potential for methanogenesis, chemoheterotrophy, and dark hydrogen oxidation. Spearman correlation analysis showed that genera such as *Candidatus competibacter*, *Geobacter*, *Syntrophobacter*, *Methanocella*, and *Methanosarcina* may serve as potential indicators of eutrophication. Overall, this study provides insight into the distinct roles that rare and abundant taxa play in the littoral sediments of mesotrophic plateau lakes.

## 1. Introduction

Eutrophication is a serious threat to the health of lacustrine ecosystems worldwide, reducing aquatic biodiversity and affecting the ecological function of lakes [1]. Microorganisms are essential components of lake ecosystems that play important roles in biogeochemical processes by participating in energy flows, materials cycling, elemental transformation, and pollutant biodegradation [2]. As a sink for many nutrients with the potential to cause secondary pollution of water bodies, lake sediment is an important pollution source that has become a key issue of concern in lakes [3]. The internal release of nitrogen (N) and phosphorus (P) from sediments, which is predominantly mediated by microbial communities, is the main nutrient source and one of the key causes of lake eutrophication [4], which emphasizes the importance of microbial communities in lake ecosystems.

The content and distribution of sediment nutrients are affected by sampling location and human disturbance. Littoral zones are transitional boundaries or ecotones between terrestrial and aquatic ecosystems that contain a variety of microbes, submerged plants, and animal communities, making them heterogeneous areas with higher niche diversity than pelagic zones [5]. However, littoral zones are potential hotspots for localized algal blooms and greenhouse gas emissions. For example, the contribution of the littoral zone to sediment methane emissions was found to be comparable to that of the profundal zone of Taihu Lake, despite being much smaller [6]. Similarly, littoral zones have been shown to contribute to as much degradation of organic matter in Lake Constance as the pelagic water column, despite the littoral zone’s surface area comprising less than 10% of the lake’s total surface area [7]. Therefore, understanding the littoral zone will facilitate the conservation and sustainable development of lake ecosystems. However, current knowledge regarding the microbial community of sediments in plateau lake littoral zones remains scarce.

Microorganisms are important drivers in the cycling of carbon (C), N, and P in aquatic ecosystems [8,9], and ecosystem function can be affected by the diversity, composition, and interactions of microbial communities [10]. Microbial community structure in sediments always changes with environmental variables (e.g., organic matter content, nutrient concentration, pH, salinity, etc.); thus, shifts in microbial functional groups influence elementary cycles [11,12]. Many studies have shown that microbial community structure can be used as an indicator of lake trophic status because of the quick and strong response of microbes to environmental stress [13,14]. Under normal conditions, microbial communities are expected to consist of a few abundant taxa and a large number of rare taxa in natural ecosystems [15,16]. Taxa with distinct abundance patterns are likely to have different ecological functions and environmental responses. Previous studies have demonstrated that abundant taxa drive major functions (e.g., carbon metabolism and nutrient cycling) in ecosystems because of their high numbers, whereas rare taxa tend to be involved in multi-functionality [16,17]. Moreover, recent studies have indicated that rare bacteria have broader environmental adaptability and can be considered as seed banks [18,19]. Many studies have elucidated the diversity, distribution, ecological functions, and environmental responses of abundant and rare taxa in different ecosystems, such as lakes [20], hot springs [21], shallow coastal sediments [22], and agricultural soils [23]. Notably, Zhao et al. (2022) highlighted that abundant taxa are more significantly impacted by eutrophication than their rare counterparts, which may have crucial implications for the structure and function of planktonic fungal communities in marine ecosystems [24]. Eutrophication was identified as a key driver of homogenization within bacterial and fungal communities, with generalist fungal species exerting a more pronounced effect on beta diversity across trophic gradients than specialist species [25]. This distinction underscores the importance of examining these sub-communities separately to unravel the mechanisms behind variations in microbial diversity in response to eutrophication. However, few studies of plateau lake ecosystems have focused on the roles of different taxa for both bacteria and archaea. A better understanding of the mechanisms that influence highly connected taxa composition and structure may provide insight into the underlying response of the whole community.

Plateau lakes are important aquatic ecosystems that are sensitive to anthropogenic disturbances due to their poor water exchange ability; accordingly, they are considered sensitive sentinels of global climate change [26]. Erhai Lake, which is the second largest freshwater lake in the Yunnan-Guizhou Plateau region, plays a significant role in local socio-economic development issues, including drinking water supply, irrigation, tourism, and regional climate regulation. However, the lake has undergone anthropogenic eutrophication since the rapid intensification of agricultural development and urbanization around the watershed in the last few decades. Consequently, Erhai Lake is transitioning from a mesotrophic to a eutrophic state. Previous studies indicated that N and P released from sediments were serious ecological pollution risks in Erhai Lake [27,28]. Although studies have investigated the overall composition of bacterial communities in some sites within the lake, we still lack a comprehensive understanding of the community structure of both bacteria and archaea in the littoral zone of Erhai Lake. Therefore, this study was conducted to (1) determine the composition, diversity, and interactions of archaea and bacteria in the sediment and their responses to nutrient loading; (2) identify keystone species that maintain community stability; and (3) determine the potential functions of both abundant and rare archaeal and bacterial communities in littoral sediments of a mesotrophic plateau lake.

## 2. Materials and Methods

### 2.1. Site Description and Sample Collection

Erhai Lake (25°36′–25°58′ N, 100°06′–100°18′ E), the second largest fault lake in China, is located in Dali, Yunnan Province, at an elevation of 1974 m. The covering area and watershed area are about 249.8 km^2^ and 2656 km^2^, respectively. The average depth is 10.5 m with a maximum depth of 20.9 m and the reservoir capacity is approximately 2.8 × 10^9^ m^3^. The annual average climate temperature is 15 °C. There are 117 tributaries that flow into the lake, but only 1 outlet.

**Figure 1:**
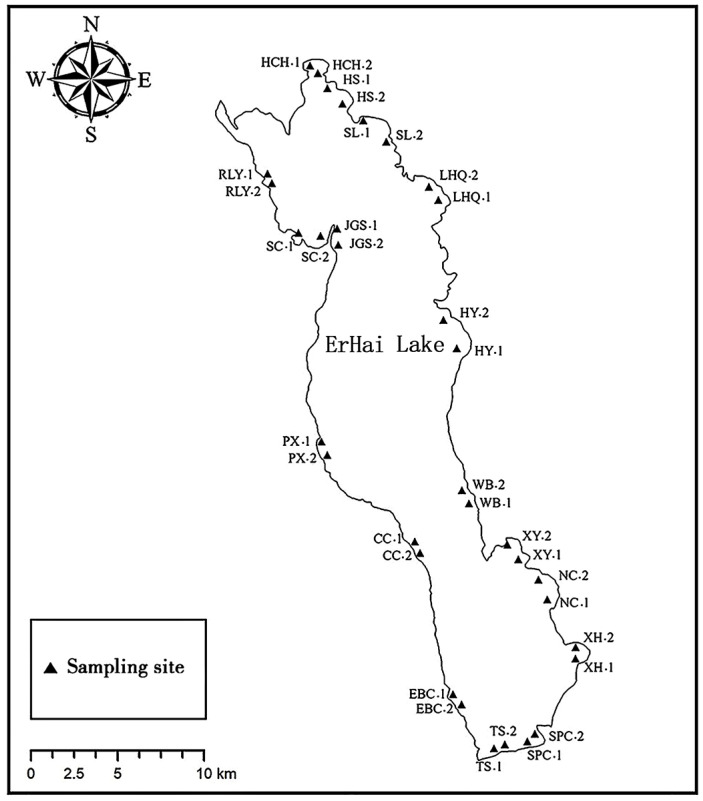
Sampling sites of Erhai Lake.

In July 2022, a total of 34 sediment samples were collected from the littoral zone along the shore of Erhai Lake (Figure 1). From each site, three random surface sediment samples (0–5 cm) were collected using core samplers and manually mixed into one sample (200 g in total) for further use. The corresponding overlying water samples were collected using plexiglass water samplers. Samples were transported to the laboratory at 4 °C, after which the sediment samples were kept at −20 °C until processed for molecular analyses and the water samples were stored at 4 °C until their physicochemical properties were analyzed.

### 2.2. Environmental Factors Analysis

The pH, dissolved oxygen (DO), and temperature (WT) of the overlying water were measured in situ during sampling using a HACH HQ40D portable multimeter. Water transparency was determined based on Secchi depth (SD). Chemical oxygen demand (COD) was measured with potassium dichromate as an oxidant using a spectrophotometer (HACH DR3900, Loveland, CO, USA). The permanganate index (PI, also known as permanganate chemical oxygen demand) was determined using the potassium permanganate method (GB11892-89). Water samples for chlorophyll a (Chl-a) analysis were first filtered through 0.45 μm glass fiber membranes, after which Chl-a was extracted with 90% acetone for >2 h at 4 °C in the dark and then measured by spectrophotometry (Chinese Environmental Standard HJ 897-2017). The total phosphorus (TP) content of the overlying water samples was determined at 700 nm using the molybdate colorimetry method after potassium sulfate (K_2_S_2_O_8_) digestion. Orthophosphate (PO_4_^3−^-P) concentration was determined by ion chromatography (ECO-IC, Metrohm, Herisau, Switzerland). Total nitrogen (TN) content was determined at 220–275 nm using UV spectroscopy after alkaline K_2_S_2_O_8_ digestion. The total dissolved nitrogen (TDN) and ammonium nitrogen (NH_4_^+^-N) of the overlying water were determined by colorimetry at 220–275 nm and using Nessler’s reagent spectrophotometry at 420 nm, respectively, after filtering samples through a 0.45 µm cellulose acetate membranes (Bikeman Bio, Changde, China). The ratios of total nitrogen to total phosphorus (N/P) were also calculated. To evaluate the trophic state of each site, the trophic state index (TSI) was calculated as described by [25].

### 2.3. DNA Extraction and PCR Amplification

Total DNA extraction from the sediment samples was performed using a FastDNA SPIN Kit for Soil (MP Biomedicals, Solon, CA, USA) according to the manufacturer’s protocols. The primer set of 341F (5′-CCTACGGGNGGCWGCAG-3′)/805R (5′-GACTACHVGGGTATCTAATCC-3′) was used for PCR amplifications of the V3-V4 regions of bacterial 16S rRNA gene. The primer set of 1106F (5′-TTWAGTCAGGCAACGAGC-3′)/1378R (5′-TGTGCAAGGAGCAGGGAC-3′) was used to amplify the V9 hypervariable region of archaeal 16S rRNA gene. The amplification conditions were set as follows: for bacteria, an initial denaturation at 94 °C for 5 min, 30 cycles of 30 s at 94 °C, 30 s at 53 °C, 30 s at 72 °C, and a final extension at 72 °C for 8 min. For archaea, an initial denaturation at 95 °C for 3 min, 30 cycles of 20 s at 95 °C, 20 s at 53 °C, 30 s at 72 °C, and a final extension at 72 °C for 5 min. PCR products were purified using the EZNA^®^ Gel Extraction Kit (Omega Bio-Tek, Winooski, VT, USA) and pooled in equimolar amounts. Then, paired-end sequencing (PE250) was performed on an Illumina Nova 6000 platform at Magigene Biotechnology Co., Ltd., Guangzhou, China, according to the standard procedures. The sequences are available as raw FASTQ files in the NCBI SRA archive under project ID PRJNA1123317.

### 2.4. Sequence Analysis

The sequences were processed using QIIME2 (version 2020.11.0). To ensure quality, fastp (version 0.14.1) was used for splicing and conducting quality control of the raw reads. Adapters and primers were trimmed off to get paired-end clean reads using Cutadapt (v1.9.1). Paired-end clean reads were processed by USEARCH (v10.0.240), including merging paired reads, filtering low-quality reads, and chimera removal. DADA2 was employed for denoising and identifying amplicon sequence variants (ASVs) [29]. The representative ASVs were annotated based on the SILVA 132 database to identify the taxonomy of each ASV. The ASVs assigned as chloroplasts and mitochondria and unknown were excluded from downstream analysis. Alpha diversity indices (chao1, Simpson, Shannon, ACE, and goods coverage) were calculated using the ‘vegan’ package in the R program. Potential metabolic functions were predicted from 16S rRNA data based on the Kyoto Encyclopedia of Genes and Genomes (KEGG) database using PICRUSt2 and the Functional Annotation tool of the Prokaryotic Taxa database (FAPROTAX).

### 2.5. Definition of Abundant and Rare Taxa

All ASVs were classified into the following six categories based on the relative abundance as described in previous studies [30,31]: (i) AAT (always abundant taxa, ASVs ≥ 1% in all samples); (ii) ART (always rare taxa, ASVs < 0.01% in all samples); (iii) MT (moderate taxa, ASVs between 0.01% and 1% in all samples); (iv) CRT (conditionally rare taxa, ASVs < 0.01% in some samples and <1% in all samples); (v) CAT (conditionally abundant taxa, ASVs ≥ 1% in some samples and >0.01% in all samples); and (vi) CRAT (conditionally rare and abundant taxa, ASVs ranging from rare (<0.01%) to abundant (≥1%)). To simplify downstream analysis, CRT and ART were regarded as rare taxa, while CRAT, AAT, and CAT were considered abundant taxa. The classification of microbial taxa yielded three groups: rare taxa (CRT and ART), abundant taxa (CRAT, AAT, and CAT), and moderate taxa (MT). Further analysis was dependent on these categories and mainly focused on rare and abundant taxa.

### 2.6. Co-Occurrence Network Analyses and Keystone Species Definition

Network analysis and identification of potential keystone taxa in the 34 samples were conducted based on Spearman correlation coefficients. To simplify the dataset for better visualization, only ASVs that were detected in more than 50% of the samples were retained for network analysis. The Spearman correlation coefficients were calculated using the ‘Hmisc’ package in the R program (Version 4.2.1). A Spearman coefficient of greater than 0.6 (or less than −0.6) and a *p*-value less than 0.05 indicated a significant correlation. Gephi was used to visualize the networks [32]. The topological roles of each node can be evaluated based on the values of within-module connectivity (Zi) and among-module connectivity (Pi). Next, the network nodes were divided into four categories: (i) peripherals (Zi < 2.5, Pi < 0.62); (ii) module hubs (Zi > 2.5, Pi < 0.62); (iii) connectors (Zi < 2.5, Pi > 0.62); and (iv) network hubs (Zi > 2.5, Pi > 0.62). Network nodes (ASVs) identified as network hubs, module hubs, and connectors were considered keystone species, which are considered to play key roles in microbial community structure and potential functions.

### 2.7. Other Statistical Analyses

The functional differences between abundant and rare taxa were compared by Statistical Analysis of Metagenomic Profiles (STAMP) [33]. Spearman’s correlation analysis was performed and visualized using the “Hmisc” and “corrplot” packages in the R software (Version 4.2.1). *p* < 0.05 was considered to identify statistically significant differences, and asterisks represented the significant levels at *p* < 0.05 (*), *p* < 0.01 (**), and *p* < 0.001 (***).

## 3. Results

### 3.1. Overall Microbial Community Compositions and Diversity

Overall, 2,850,609 high-quality bacterial and 1,927,903 archaeal sequences were obtained from 34 samples after amplicon sequencing, with 74,654–94,514 sequences being obtained per sample for bacteria and 42,464–63,136 sequences per sample for archaea. Good’s coverage was close to 1.0 for both bacterial and archaeal communities across the 34 samples, indicating that the majority of the microbial taxa had been recovered (Table S1). To equalize sequencing depth, each sample was rarefied to the lowest sequence numbers (74,654 sequences for bacteria and 42,464 sequences for archaea) across all samples and then classified into ASVs. A total of 108,543 bacterial and 6357 archaeal ASVs were identified at 100% identity. For each sample, 1981 to 5859 bacterial ASVs and 271 to 532 archaeal ASVs were obtained (Table S1). The alpha diversity indices (Shannon and Simpson) and the richness estimators (ACE and Chao 1) for both bacteria and archaea are summarized in Table S1. Most samples showed similar bacterial alpha diversity indices and richness estimators except site HCH.1, in which the alpha diversity was relatively low and the exception of HCH.1 was not observed in the archaeal community.

**Figure 2:**
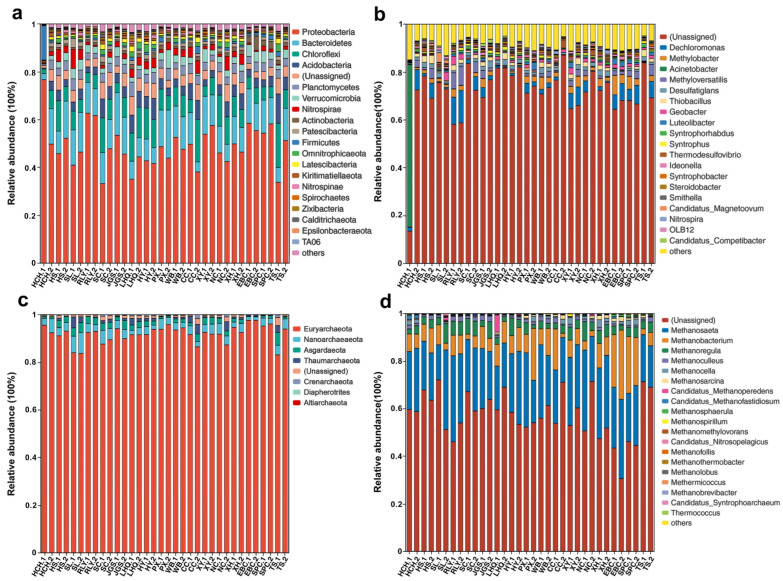
The relative abundance of (**a**) bacterial communities in sediments at the phylum and (**b**) genus level, and (**c**) the archaeal communities at the phylum and (**d**) genus level.

Evaluation of the bacterial community composition revealed a total of 58 phyla, 125 classes, 285 orders, 350 families, 569 genera, and 438 species across all 34 samples. The three most abundant bacterial phyla were Proteobacteria (33.27–76.67%), Bacteroidetes (2.50–20.10%), and Chloroflexi (1.92–18.49%), followed by Acidobacteria (0.45–2.24%), Planctomycetes (0.67–6.03%), Verrucomicrobia (0.31–7.71%), and Nitrospirae (0.44–8.04%), except at site HCH.1, where there was a high proportion of Firmicutes (14.29%) (Figure 2a). At the genus level, *Dechloromonas* (0.04–13.55%), *Methylobacter* (0.05–8.08%), *Methyloversatilis* (0.03–7.94%), and *Desulfatiglans* (0.33–2.93%) were abundant across most of the samples (Figure 2b). Interestingly, the proportion of unassigned genera ranged from 58.16% to 87.10% across most sampling sites, while the unassigned proportion was only 13.44% in HCH.1 due to its high level of *Acinetobacter* (67.84%) (Figure 2b).

Evaluation of the archaeal community revealed 7 phyla, 15 classes, 14 orders, 19 families, 21 genera, and 19 species. The most abundant archaeal phyla among the samples was Euryarchaeota (83.13–97.63%), while Nanoarchaeaeota (1.45–8.70%), Asgardaeota (0.20–5.32%), Thaumarchaeota (0.06–4.54%), Crenarchaeota (0.03–1.66%), Diapherotrites (<0.15%), and Aliarchaeota (only at sites XY.2 and EBC.1 with a relative abundance < 0.10%) were also found (Figure 2c). The most abundant genera was *Methanosaeta* (14.76–36.18%), followed by *Methanobacterium* (1.74–29.11%), *Methanoregula* (1.53–8.33%), *Methnoculleus* (0.40–2.38%), *Methnocella* (0.13–3.21%), and others (Figure 2d).

### 3.2. Identification of Abundant and Rare Sub-Communities

**Figure 3:**
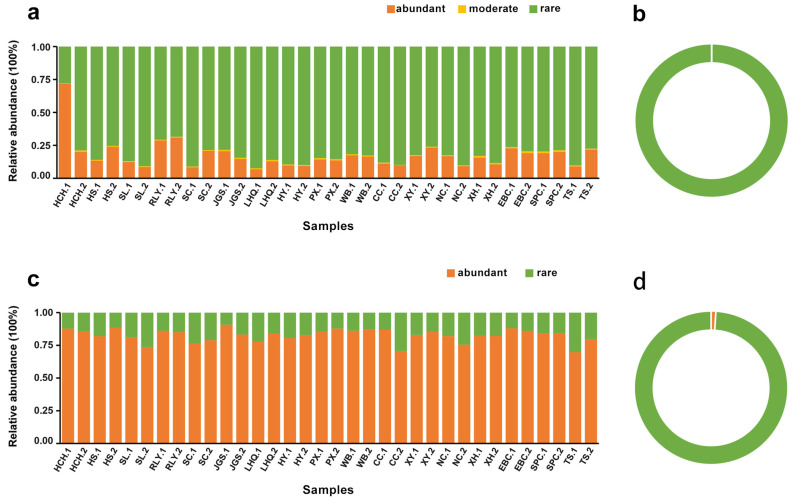
Relative abundance and composition of sub-communities to the overall sequences or total ASVs. (**a**) Relative abundance of bacterial sub-communities; (**b**) Composition of bacterial ASVs; (**c**) Relative abundance of archaeal sub-communities; (**d**) Composition of archaeal ASVs.

All microbial taxa were divided into six categories and then simplified into three main categories for further analysis (Figure 3, Table 1). A summary of the classifications for the total 108,543 bacterial ASVs revealed that 10 and 30 ASVs were considered CAT and CRAT, respectively, while no ASVs were categorized as AAT. The total number of abundant taxa for ASVs was 40 with an average relative abundance of 17.63%. Only six bacterial ASVs were classified into MT, contributing 0.85% of the average relative abundance. The ASV number for rare taxa was 108,497, representing 81.52% of the average relative abundance in each sample. Specifically, ART and CRT comprised 88,143 and 20,354 ASVs, respectively, contributing 10.82% and 70.70% of the average relative abundance per sample. In contrast to the bacterial community, 64 out of 6357 archaeal ASVs affiliated as abundant taxa contributed 82.70% of the average relative abundance in each sample. Interestingly, two archaeal ASVs (ASV1 and ASV2, affiliated to Order Methanomicrobiales) belonging to AAT had a high abundance (42.85%), suggesting that potential methanogenesis was widespread in the sediment of Erhai Lake. The remaining 6293 of 6357 archaeal ASVs, which were regarded as rare, contributed only 17.30% to the average relative abundance in each sample. No ASV was divided into MT for archaea. These results implied that rare species dominated the bacterial community, while abundant taxa were predominant in the archaeal community within Erhai Lake littoral sediments.

### 3.3. Metabolic Potential of Abundant and Rare Sub-Communities

**Figure 4:**
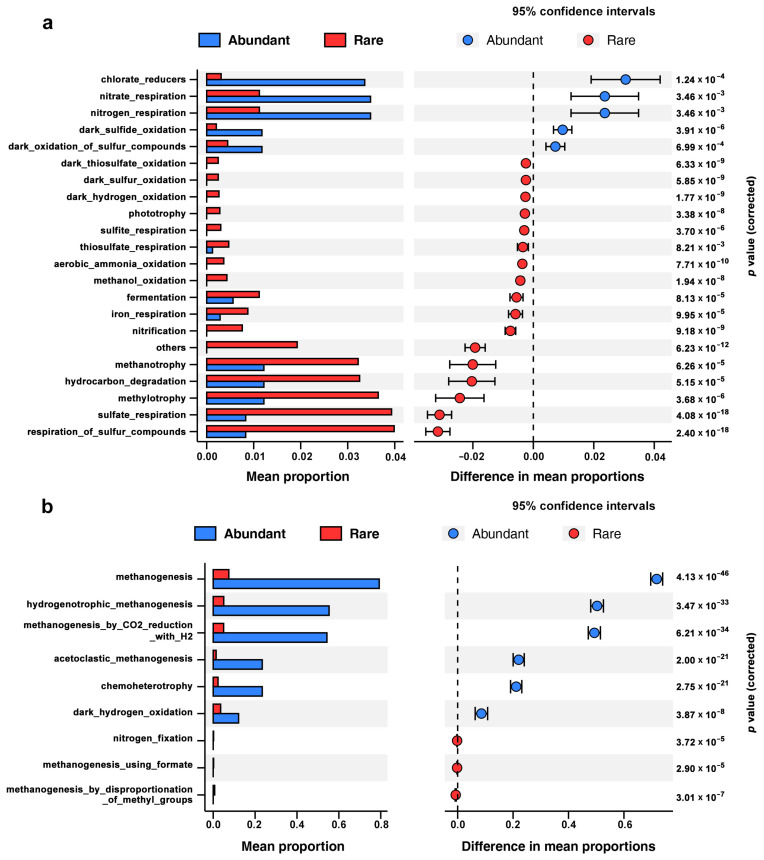
Difference of functional pathways between abundant and rare sub-communities in littoral sediments from Erhai Lake. (**a**) Bacteria; (**b**) Archaea.

To compare the metabolic potential of abundant and rare sub-communities, FAPROTAX and PICRUSt2 analyses were performed. The results showed that abundant bacterial sub-communities were predicted to have a higher abundance of functional pathways involved in nitrogen cycles (e.g., nitrate and nitrogen respiration), sulfur cycles (e.g., dark sulfide oxidation and dark oxidation of sulfur compounds), and chlorate reducers than other pathways (Figure 4a). Additionally, rare bacterial sub-communities had more sequences annotated to carbon cycles and other functional pathways, such as methanotrophy, hydrocarbon degradation, and sulfate respiration. For archaeal communities, methanogenesis was the main potential ecological function involving different trophic types. Archaeal-abundant sub-communities contained a much higher abundance of functional pathways associated with methanogenesis, chemoheterotrophy, and dark hydrogen oxidation than other pathways (Figure 4b). Surprisingly, some functional pathways were only assigned in rare sub-communities. For example, dark thiosulfate oxidation, dark sulfur oxidation, dark hydrogen oxidation, phototrophy, sulfite respiration, and aerobic ammonia oxidation were detected only in rare bacterial sub-communities. Additionally, nitrogen fixation, methanogenesis using formate, and methanogenesis by disproportionation of methyl groups only presented in archaeal rare sub-communities, but not in abundant sub-communities. PICRUSt2 functional prediction predicted more KEGG functions in rare sub-communities than abundant sub-communities for both bacteria and archaea (Table 2). These findings indicated that rare sub-communities were more diverse in function and different sub-communities contributed differently to specific metabolic pathways.

### 3.4. Overall Microbial Network Co-Existence Patterns

**Figure 5:**
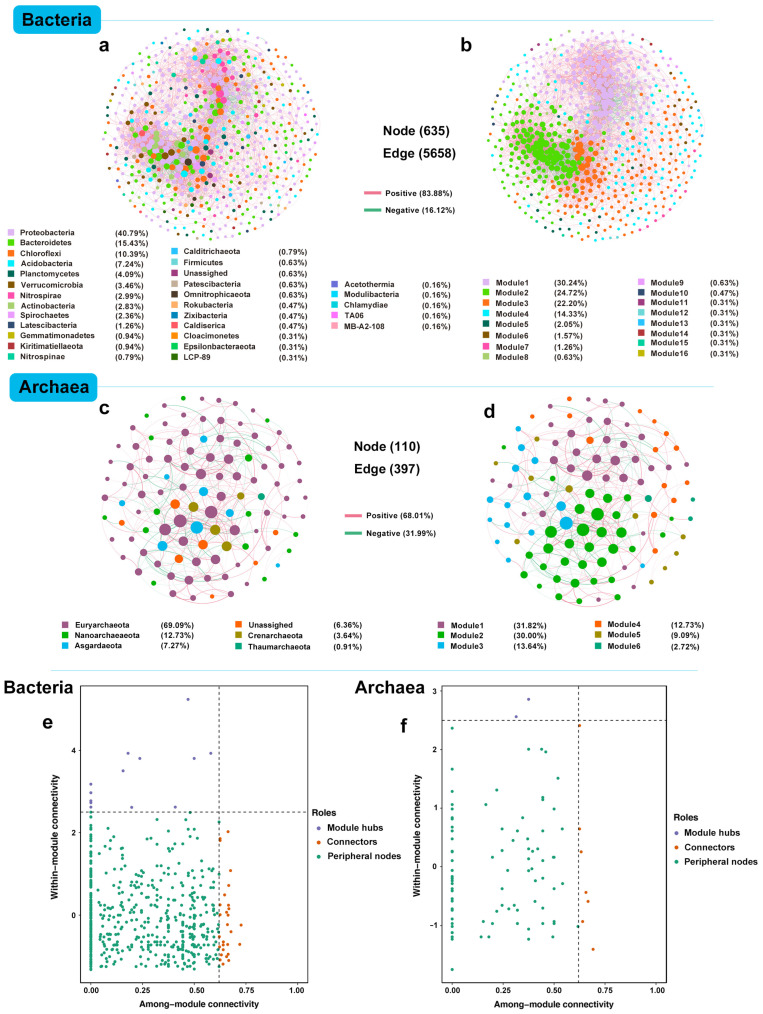
Overall co-occurrence networks and Zi-Pi plot of the bacterial and archaeal communities based on pairwise Spearman’s correlations between ASVs. The nodes were colored according to different types of (**a**,**c**) phylum and (**b**,**d**) modularity classes, respectively. A connection stands for a strong (Spearman r > 0.6 or r < −0.6) and significant (*p* value < 0.05) correlation. For each panel, the size of each node is proportional to the number of connections (i.e., degree). The green and pink edges indicate negative and positive interactions between two individual nodes, respectively. Zi-Pi plot showing the distribution of (**e**) bacterial and (**f**) archaeal ASVs based on their topological roles. Each symbol represents an ASV. The topological role of each ASV was determined according to the scatter plot of within-module connectivity (Zi) and among-module connectivity (Pi). (i) network hubs: nodes with Zi > 2.5 and Pi > 0.62; (ii) module hubs: nodes with Zi > 2.5 and Pi ≤ 0.62; (iii) connectors: nodes with Zi ≤ 2.5 and Pi > 0.62; and (iv) peripheral nodes: nodes with Zi ≤ 2.5 and Pi ≤ 0.62.

The correlation-based network consisted of 635 nodes (ASVs) with 5658 edges (correlations) for bacteria and 110 nodes with 397 edges for archaea (Figure 5). Overall, taxa tended to co-occur (positive correlations, pink lines) rather than co-exclude (negative correlations, green lines). Positive correlations accounted for 83.88% and 68.01% of the potential interactions in bacterial and archaeal networks, respectively, whereas negative correlations accounted for 16.12% and 31.99% of the interactions for bacterial and archaeal co-existence patterns. Considering all correlations, the links between bacteria were more complex than those between archaea, indicating that potential interactions are stronger in bacterial networks. Nodes in the bacterial network mainly belonged to Proteobacteria (40.79%), Bacteroidetes (15.43%), Chloroflexi (10.39%), Acidobacteria (7.24%), Planctomycetes (4.09%), Verrucomicrobia (3.46%), and Nitrospirae (2.99%) (Figure 5a). Furthermore, bacterial networks were clearly parsed into 16 modules (a module is defined as a group of ASVs that are linked more tightly together). Of these, the major modules (1, 2, 3, 4, and 5) accounted for 30.24%, 24.72%, 22.20%, 14.33%, and 2.05% of the entire bacterial network, respectively (Figure 5b). The nodes in the archaeal network were primarily occupied by Euryarchaeaeota (69.09%), Nanoarchaeaeota (12.73%), and Asgardaeota (7.27%) (Figure 5c). Only six modules were clustered for archaeal networks, with major modules 1 to 5 representing 31.82%, 30.00%, 13.64%, 12.73%, and 9.09% of the entire archaeal network, respectively (Figure 5d). Among these modules, the taxonomic compositions varied, with Proteobacteria, Bacteroidetes, Chloroflexi, Nitrospirae, Acidobacteria, and Euryarchaeota being dominant for bacterial and archaeal modules, respectively (Figure S1).

Zi-Pi analysis was performed to determine the potential keystone taxa within each network. Based on the Zi-Pi plot, 41 bacterial ASVs and 9 archaeal ASVs were defined as keystone taxa and Proteobacteria and Euryarchaeota were the most prominent module hubs and connectors for bacteria and archaea, respectively (Figure 5e,f and Figure S2, Tables S2 and S3). The bacterial keystone taxa included taxa from the classes Gammaproteobacteria (9 ASVs), Deltaproteobacteria (8 ASVs), Bacteroidia (6 ASVs), Thermodesulfovibrionia (Nitrospirae, 1 ASV), Subgroup_6 (Acidobacteria, 1 ASVs), Subgroup_22 (Acidobacteria, 1 ASVs), Dehalococcoidia (Chloroflexi, 3 ASV), Anaerolineae (Chloroflexi, 2 ASVs), Verrucomicrobiae (3 ASVs), Planctomycetacia (2 ASVs), and Phycisphaerae (Planctomycetes, 1 ASVs). Additionally, two ASVs were assigned as Chloroflexi/LCP_89, while the other two ASVs were unassigned (Table S2, Figure S2). In the archaeal network, the keystone species included taxa from the classes Woesearchaeia (Nanoarchaeaeota, 2 ASVs), Methanomicrobia (Euryarchaeota, 4 ASVs), Thermoplasmata (Euryarchaeota, 2 ASVs), and Lokiarchaeia (Asgardaeota, 1 ASV) (Table S3). Keystone taxa spanned a range of relative abundances in the overall communities (0.02% to 0.62% for bacteria and 0.03% to 0.86% for archaea). Most of the bacterial keystone taxa (38 of 41 ASVs) were classified as rare (Table S2), while over half of the archaeal keystone taxa (5 out of 9 ASVs) were abundant (Table S3). This highlights the significant roles of rare taxa in bacterial communities and abundant taxa in archaeal communities.

### 3.5. Correlation between Microbial Community and Environmental Factors

The physicochemical properties of the overlying water are shown in Table S4. The TN and TP ranged from 0.526 to 0.677 mg/L and 0.024 to 0.037 mg/L, respectively. Additionally, the calculated TSI ranged from 42 to 49, indicating the water samples were mesotrophic.

The Spearman correlations between physicochemical properties and alpha diversity indices of the total microbial communities are shown in Figure S3. The ACE and Chao 1 indices of the overall bacterial community were significantly negatively correlated with TP (*p* < 0.05, Figure S3a), implying that P nutrient concentrations may affect bacterial community richness in the littoral sediments of Erhai Lake. Notably, no significant correlation was observed between the identified environmental variations and the diversity indices of the archaeal community (Figure S3b).

**Figure 6:**
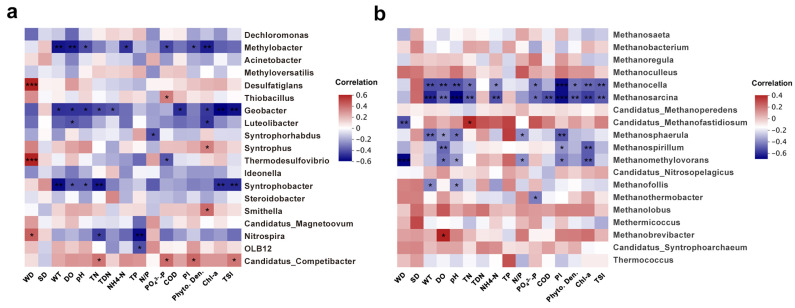
Heatmaps showing Spearman correlation between environmental factors and microbial species of (**a**) bacterial and (**b**) archaeal communities at genus level. Blue indicates a negative correlation, while red indicates a positive correlation. *** *p* < 0.001, ** *p* < 0.01, * *p* < 0.05.

To further understand the response of bacteria and archaea to environmental variations, the dominant bacterial and archaeal communities were selected and their responses to environmental factors were analyzed (Figure S3c,d and Figure 6a,b). The results showed that Proteobacteria, Verrucomicrobia, Nitrospirae, Actinobacteria, Patescibacteria, Firmicutes, Zixibacteria, Calditrichaeota, Epsilonbacteraeota, and TA06 were significantly correlated with water depth, water transparency, WT, DO, pH, NH_4_^+^-N, TP, N/P ratio, PO_4_^3−^-P, COD, permanganate index, phytoplankton density, and Chl-a (Figure S3c). Additionally, the NH_4_^+^-N and TP contents and permanganate index were positively correlated with the relative abundance of Nanoarchaeota and Asgardaeota, but negatively related with that of Euryarchaeota (Figure S3d). At the genus level, many genera responded negatively to environmental factors with *Geobacter*, *Syntrophobacter*, *Methanocella*, and *Methanosarcina* being significantly negatively correlated to TSI, while *Candidatus Competibacter* showed a positive response (Figure 6a,b).

To verify the role those environmental variables played in the abundances of keystone taxa, Spearman correlation analysis was conducted (Figure S3e,f). Generally, the results showed that ASV_97 (genera *Candidatus Competibacter*, Proteobacteria), ASV_433 (Gammaproteobacteria), ASV_496 (genera *Candidatus Competibacter*, Proteobacteria), ASV_826 (Bacteroidetes), and ASV_429 (Class Anaerolineae, Chloroflexi) were positively influenced by TN, NH_4_^+^-N, PI, DO, pH, COD, PO_4_^3−^-P, and Chl-a, while these environmental variables negatively affected the keystone taxa of ASV_732 (genera *Methyloparacoccus*, Proteobacteria), ASV_1179 (Verrucomicrobia), and ASV_1207 (Planctomycetes). Moreover, TSI was significantly positively related to ASV_97, ASV_433, ASV_496, ASV_429, and ASV_826, whereas it was negatively influenced by ASV_1179, implying the potential indicator roles of these keystone species in the eutrophication of Erhai Lake. Evaluation of archaeal keystone taxa revealed that most were positively correlated with environmental factors, except that ASV_47 (genera *Methanobacterium*, Euryarchaeota) was significantly negatively related to PI, DO, and WT. No significant correlation was found between ASV_21 (genera *Methanosaeta*, Euryarchaeota) and any of the detected environmental variables. Notably, only ASV_88 (genera *Methanobacterium*, Euryarchaeota) had a positive relationship with TSI. These significant correlations between keystone taxa and water nutrients or environmental factors suggest that these key species play important roles in the eutrophication of lakes.

## 4. Discussion

Lake sediment ecosystems harbor complex microbial communities that are involved in a variety of biogeochemical transformations. Microbial communities respond quickly to anthropogenic disturbances and are sensitive indicators of the water quality status of lakes [13,31]. Different abundance patterns exist within microbial communities, such as the presence of abundant or rare taxa, shaping complex interaction networks and playing discrepant ecological roles in sediments [34,35]. However, little is known about the littoral sediments of mesotrophic plateau lakes. In this study, we investigated the diversity, community composition, potential functions, and co-occurrence patterns of bacterial and archaeal communities, as well as the related environmental factors in the littoral sediments of mesotrophic Erhai Lake on the Yunnan–Guizhou Plateau with a high sampling coverage to draw a comprehensive picture of these ecosystems.

### 4.1. Characteristics of the Overall Microbial Community

The dominant phyla identified in this study have been widely recognized as prevalent in freshwater lake ecosystems worldwide, playing a pivotal role in C and N metabolism [2]. The predominance of Proteobacteria and Chloroflexi in the littoral sediments of this lake was similar to that observed in previous studies of various lakes of different trophic status [36]. At the genus level, the main bacterial genera were *Dechloromonas*, *Methylobacter*, *Acinetobacter*, *Methyloversatilis*, *Desulfatigians,* and *Thiobacillus*, which have the potential functions of nitrogen, sulfur, and aromatic pollutants metabolism as well as methane oxidation. Notably, unlike at other sites, *Acinetobacter* (affiliated with Gammaproteobacteria) occupied a relatively high abundance at site HCH.1. *Acinetobacter* is a well-known hydrocarbon degrader, especially with respect to aromatic compounds and alkanes [37]. The high proportion of *Acinetobacter* at site HCH.1 might demonstrate a unique pollution condition at this site. If so, this would indicate *Acinetobacter* could be a useful indicator. Firmicutes, which is generally ubiquitous in aquatic ecosystems, but a minor member of freshwater lake ecosystems [38], also showed a relatively high abundance at site HCH.1. Previous studies have reported that Firmicutes can grow chemo-organotrophically on a variety of organic substrates and degrade many types of organic pollutants, such as petroleum hydrocarbons [39], polychlorinated biphenyl [40], and hexahydro-1,3,5-trinitro-1,3,5-triazine [41]. The abundance of these bacteria implies that there is organic pollution near site HCH.1.

Among archaea, Euryarchaeota was identified as the most influential phylum, with a relative abundance >80%. Euryarchaeota have been found to be predominant in some lake sediments, such as those of Taihu Lake [42], Dianchi Lake [43], and Geneva Lake [44]. Some previous studies have also indicated that archaeal community diversity varies with sampling depth in lake sediments and water columns [45,46]. Archaea are widely known for their roles in numerous biogeochemical processes, including ammonia oxidation, methanogenesis, and sulfate reduction [47]. Euryarchaeotes are mainly known as methanogens, although some may also degrade hydrocarbons and be involved in sulfur, nitrogen, and iron cycling [47,48]. At the genus level, the three most abundant genera (*Methanosaeta*, *Methanobacterium,* and *Methanoregula*) were all methanogens, revealing that sediments in the littoral zones of Erhai Lake are potential hotspots for CH_4_ emissions. *Methanosaeta* utilizes acetate as a substrate, while *Methanobacterium* and *Methanoregula* are hydrogenotrophic methanogens [49]. Additionally, a small number of methylotrophic methanogens (e.g., *Methanomethylovorans* and *Methanolobus*) were also detected, demonstrating a high metabolic diversity of methanogens in these sediments.

### 4.2. Rare and Abundant Taxa Dominated Co-Occurrence Networks

Network analysis has been successfully applied to explore co-occurrence patterns and interspecific interactions of microbial communities in various ecosystems. In this study, more positive correlations than negative correlations were observed in the networks of both bacteria and archaea. The enhancement of positive interactions among bacteria and archaea in this study may occur because of the higher availability of nutrients and bioavailable carbon sources. This increased availability could reduce the competitive pressure among microbial communities, potentially leading to a less stable network in this ecosystem [50,51].

The co-occurrence networks of Erhai Lake indicate that rare bacterial taxa and abundant archaeal taxa may play irreplaceable roles in shaping their respective networks within the littoral sediments of this mesotrophic plateau lake. Similar findings were reported in a recent study of oil reservoirs [52]. Previous research has primarily focused on either rare bacterial communities or rare eukaryotic plankton communities, and the results have confirmed the significance of rare species in the overall community structure and ecological functions. For instance, Xue et al. (2018) found that most of the keystone species of eukaryotic plankton communities were affiliated with rare taxa following a reservoir cyanobacterial bloom [31]. However, our study indicated that, while rare taxa played a key role in the bacterial community, the archaeal community was dominated by abundant species. Growing evidence from various ecosystems has highlighted the importance of keystone species in co-occurrence networks, in which some keystone taxa are the drivers of the structure and function of the overall microbial community and the disappearance of such keystone taxa can affect the entire community structure and functions.

### 4.3. Rare and Abundant Taxa Play Different Potential Functions

Typically, abundant taxa contribute the majority of the overall abundance, while rare taxa account for a smaller proportion of all species’ abundance, although with a large number of ASVs. However, in our study, the contribution of rare bacterial taxa to the overall abundance was large (81.52%), which is similar to the results reported for sediments of aquaculture pond ecosystems [53]. In contrast, the archaeal community exhibited the opposite trend, with a small number of abundant taxa ASVs contributing up to 82.70% of the overall abundance. These results demonstrated that rare and abundant bacterial and archaeal sub-communities contributed differently to the overall community abundance in littoral sediments of a mesotrophic plateau lake. Specifically, abundant taxa were ubiquitous and possessed higher adaptability to environmental variations, whereas rare taxa were restricted in their distribution across samples and were stronger competitors within narrow niche breadths.

Our results showed that both rare and abundant sub-communities made different contributions to defined metabolic pathways, with rare sub-communities exhibiting greater functional diversity. Several recent studies have emphasized the paramount importance of rare taxa to ecological functions in ecosystems [18,22,53]. Rare taxa may harbor more diverse functional genes involved in C, N, P, and S cycles than abundant taxa and perform distinct or supplementary functions that contribute to the preservation of community function [16,31]. Moreover, rare taxa have been reported to be more competitive than abundant taxa within a narrow niche breadth and to preferentially metabolize some specific substrate, whereas abundant taxa have a broader niche breadth and are more adaptable to various habitats [54]. Rare taxa are important contributors to microbial diversity that increase the functional redundancy within the community [17,34]. A previous study revealed that rare sub-communities played a dominant role in shaping the complexity of bacterial ecological networks and could indirectly enhance the functionality of abundant taxa, highlighting the disproportionate roles of rare taxa in the overall communities [55].

Modularity is believed to reflect interspecies interactions and niche differentiation, with different modules potentially playing different functions. This was supported by the results of the present study, which showed the taxonomic compositions of each module differed (Figure S1). Specifically, the compositions of abundant and rare communities differed and contributed uniquely to ecological functions [21]. Moreover, keystone taxa may impact the metabolic function of entire communities, regardless of their low abundance [56]. The keystone taxa identified in our study were reportedly involved in mediating carbon, sulfur, iron, and nitrogen cycles. Consistent with previous studies, the bacterial phyla Proteobacteria (Gammaproteobacteria and Deltaproteobacteria) were predominant as keystone taxa in freshwater ecosystems. For instance, Proteobacteria are ubiquitous in aquatic habitats and play key roles in nitrogen fixation, dissimilatory nitrate reduction, denitrification, and nutrient cycling [57]. Additionally, Gammaproteobacteria and Deltaproteobacteria are recognized as dominant groups in eutrophic lakes [58,59], while Bacteroidetes have been found to be associated with cyanobacterial blooms in freshwater ecosystems [60]. Members of the phylum Verrucomicrobia act as polysaccharide degraders and play a potential role in carbon cycling in freshwater systems [61]. Chlorofexi and Nitrospirae have demonstrated diverse anaerobic metabolic capabilities and are likely involved in sulfur and nitrogen cycling [62]. The archaeal keystone ASVs affiliated with the classes Methanomicrobia, Woesearchaeia, and Thermoplasmata have the potential for carbon metabolism, sulfate reduction, and organic matter decomposition. In summary, the intricate interplay between rare and abundant taxa in the sediments of mesotrophic lakes reveals a complex ecosystem dynamic, with each group exerting unique and complementary influences on the microbial community’s metabolic potential and overall biogeochemical functioning.

### 4.4. Effects of Environmental Factors and Potential Microbial Indicators of Lake Eutrophication

In lake ecosystems, environmental factors affect the diversity, composition, and distribution of microorganisms. In our study, Spearman correlation analysis of the bacterial community showed that richness estimators (ACE and Chao1) were significantly negatively related to TP, while no detected environmental parameters were significantly related to the diversity index of the archaeal community. A recent meta-analysis of Chinese lakes revealed that the Shannon diversity of sedimental bacterial community decreased significantly with latitude and nitrate (NO_3_^−^-N), but increased with TN, TP, and total organic carbon (TOC) at the national scale [63]. Overall, these findings indicate that local environmental variations make greater contributions to shaping indigenous microbial communities at smaller spatial scales. For example, the dominant community in Hulun Lake was strongly affected by temperature, pH, DO, EC, NH_4_^+^-N, TP, TN, and COD [64]. Moreover, many low-latitude freshwater lakes such as Chaohu Lake and Dianchi Lake were influenced by nutrient physiochemical parameters (e.g., NH_4_^+^-N, NO_3_^−^-N, TN, and TP) [30,59]. The key driving environmental factors of microbial communities can reflect the main nutrient status of the lake ecosystems and provide hints for controlling lake eutrophication.

Interestingly, *Candidatus Competibacter* was found to be strongly positively related to TSI, likely because it can compete with polyphosphate-accumulating organisms (PAO) for resources. This competition may reduce the capacity for P removal [65]. In contrast, *Syntrophobacter*, a member of the class Deltaproteobacteria, was negatively correlated with TSI. This genus, which is known for anaerobic respiration, can oxidize propionate using sulfate or fumarate as an electron acceptor [66]. Given that propionate is a significant precursor of methane production [66], it is speculated that *Syntrophobacter* might directly or indirectly participate in the conversion of organic matter degradation to methane emissions, thereby reducing available carbon nutrients in lakes and negatively affecting eutrophication indices. The genus *Geobacter* has been reported to be involved in iron reduction, nitrate-dependent Fe (II) oxidation, and pollutant degradation [67]. *Geobacter* can coexist with methanogens and accelerate the production of CH_4_ by interspecies electron transfer [68]. However, *Geobacter* can also inhibit CH_4_ production by directly competing with methanogens for acetate [69]. The specific role of *Geobacter* in Erhai Lake requires further exploration. Notably, the negative correlations between the archaeal genera *Methanocella* and *Methanosarcina* and TSI indicated these microbes may serve as potential indicators. Methanogenic microorganisms have been reported to be associated with cyanobacterial blooms [70]. The significant correlations between the identified key species and TSI or other key environmental factors demonstrate that these key species play important roles in the eutrophication processes of lakes and deserve further exploration.

## 5. Conclusions

We found distinct patterns of abundance and functionality among both bacterial and archaeal taxa, highlighting the complex interactions between these microorganisms and their environment. The microbial community structure in the littoral sediments of Erhai Lake was characterized by a dominance of Proteobacteria, Bacteroidetes, Chloroflexi, and Euryarchaeota. Rare bacterial taxa were often keystone species, exerting a disproportionate influence on network structure and function. In contrast, archaeal communities were primarily driven by abundant taxa, indicating a different ecological dynamic between these two domains. This divergence in communities demonstrated the importance of considering both rare and abundant taxa when studying microbial communities and their responses to environmental changes. This study shed light on the nuanced relationships between microbial communities and their environment in mesotrophic lake sediments. Identification of keystone taxa and developing an understanding of their ecosystem roles will provide valuable information that will be useful in the development of targeted strategies to mitigate eutrophication and preserve the health of these vital aquatic ecosystems.

Although microbial indicators hold potential for application in lake restoration, the vast diversity and complex interactions within microbial communities can make it challenging to predict their responses to environmental changes or interventions. Furthermore, the utilization of microbial indicators for eutrophication prediction or the ecological management of eutrophic lakes through the regulation of specific functional microbial consortia in future commercial applications must take into account the principle of adapting to local conditions. This is due to the unique structure of microbial communities in each lake, necessitating a management approach that is specific to each lake. Further research is needed to focus on key functional microbes within plateau lakes, integrating new technologies (such as metagenomic or metatranscriptomic techniques) with traditional cultivation and isolation methods to functionally validate these key microbes. It is essential to explore the metabolic coupling among the cycles of carbon, nitrogen, phosphorus, and sulfur, and to elucidate their roles in the transformation of nutrients in eutrophic lakes.

## Figures and Tables

**Table 1:** The contribution of each taxa category to the bacterial and archaeal communities in the 34 samples at 100% identity level.

			Bacteria		Archaea	
			ASVs Number	Avg. Relative Abundance	ASVs Number	Avg. Relative Abundance
	All ASVs		108,543	100.00%	6357	100.00%
Abundant taxa	Always abundant taxa (AAT)		0	0.00%	2	42.85%
	Conditionally abundant taxa (CAT)		10	9.93%	23	29.17%
	Conditionally rare and abundant taxa (CRAT)		30	7.71%	39	10.68%
	Total		40	17.63%	64	82.70%
Rare taxa	Always rare taxa (ART)		88,143	10.82%	3565	0.58%
	Conditionally rare taxa (CRT)		20,354	70.70%	2728	16.72%
	Total		108,497	81.52%	6293	17.30%
	Moderate taxa (MT)		6	0.85%	0	0.00%

**Table 2:** Comparison of numbers of functional pathways among abundant sub-community, rare sub-community, and whole community in Erhai Lake.

	Predicted KEGG-Annotated Genes		
	Abundant Sub-Community	Rare Sub-Community	Overall Community
Bacteria	3611	7481	7481
Archaea	3701	4064	4064

## Data Availability

All raw sequence data obtained in this study are available on the Sequence Read Archive database of the National Center for Biotechnology Information under accession number PRJNA1123317.

## Supplementary Materials

The following supporting information can be downloaded at: https://www.mdpi.com/article/10.3390/microorganisms12081617/s1, Figure S1: Histograms show the taxonomy composition of the dominant modules at the phylum level; Figure S2: Taxonomic composition of bacterial keystone taxa at the phylum level; Figure S3: Heatmap showing Spearman correlation between the alpha diversity index, microbial communities and environmental factors; Table S1: Alpha diversity index of bacterial and archaeal communities in littoral sediments of Erhai Lake; Table S2: Lists of keystone taxa in co-occurrence network of bacteria; Table S3: Lists of keystone taxa in co-occurrence network of archaea; Table S4: Physicochemical characterization of samples in the littoral sediments of Erhai Lake.
