# Supplementary material for: Bacterial and Archaeal Communities in Erhai Lake Sediments: Abundance and Metabolic Insight into a Plateau Lake at the Edge of Eutrophication
Source: Microorganisms. 2024 Aug 8;12(8):1617. doi: 10.3390/microorganisms12081617 (PMC11356345; doi:10.3390/microorganisms12081617)
Supplement: Supplementary file 1 [file microorganisms-12-01617-s001.zip › Suporting information_Figures_revision.pdf]

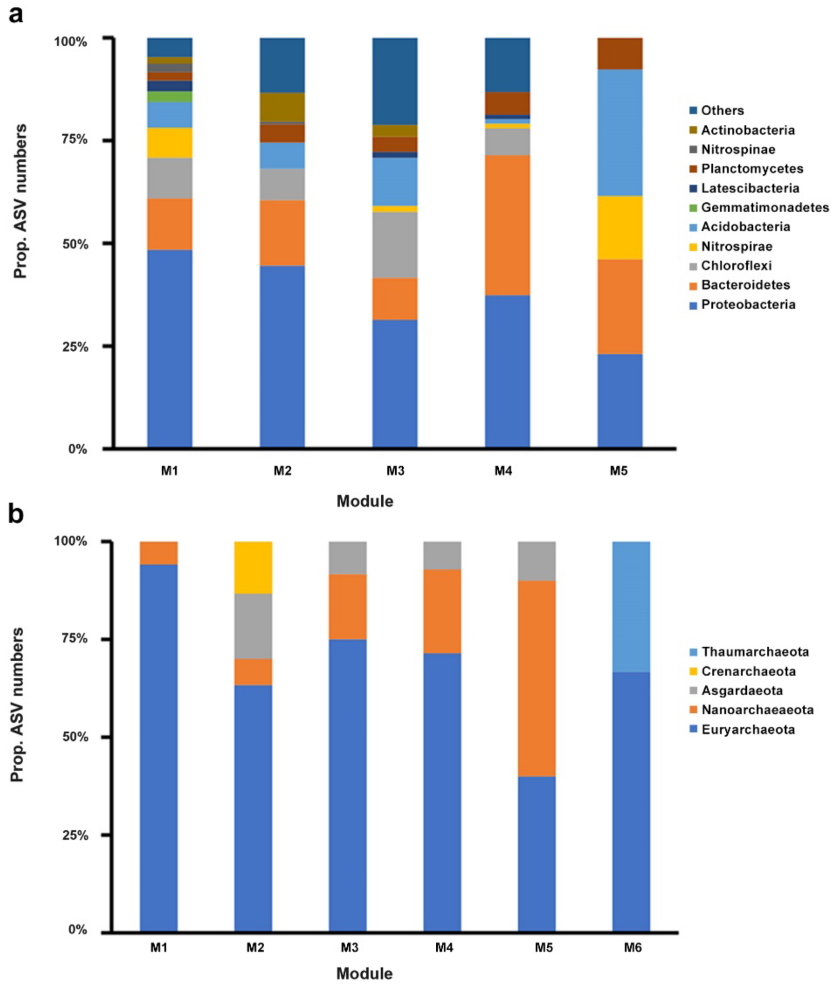

Figure S1. Histograms show the taxonomy composition of the dominant modules at the phylum level. **(a)** Bacteria; **(b)** Archaea.

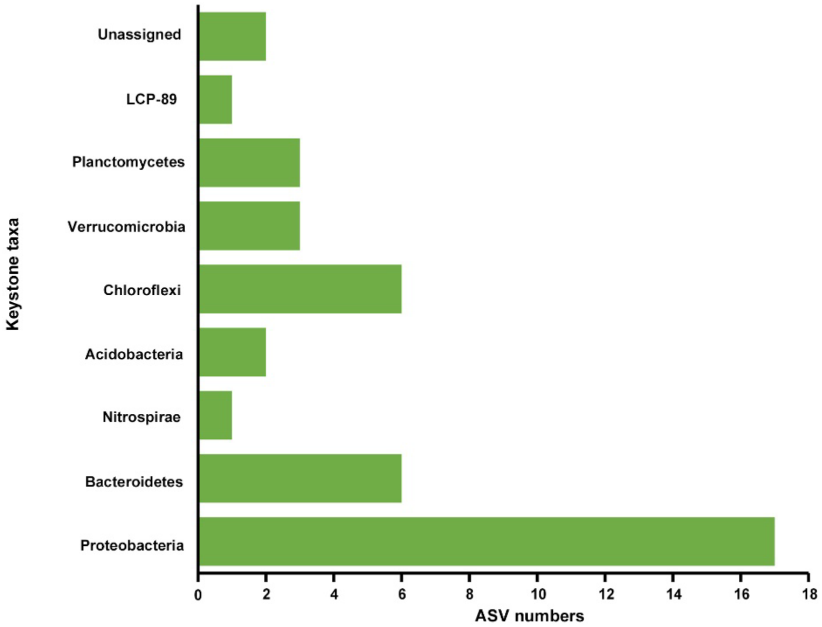

Figure S2. Taxonomic composition of bacterial keystone taxa at the phylum level.

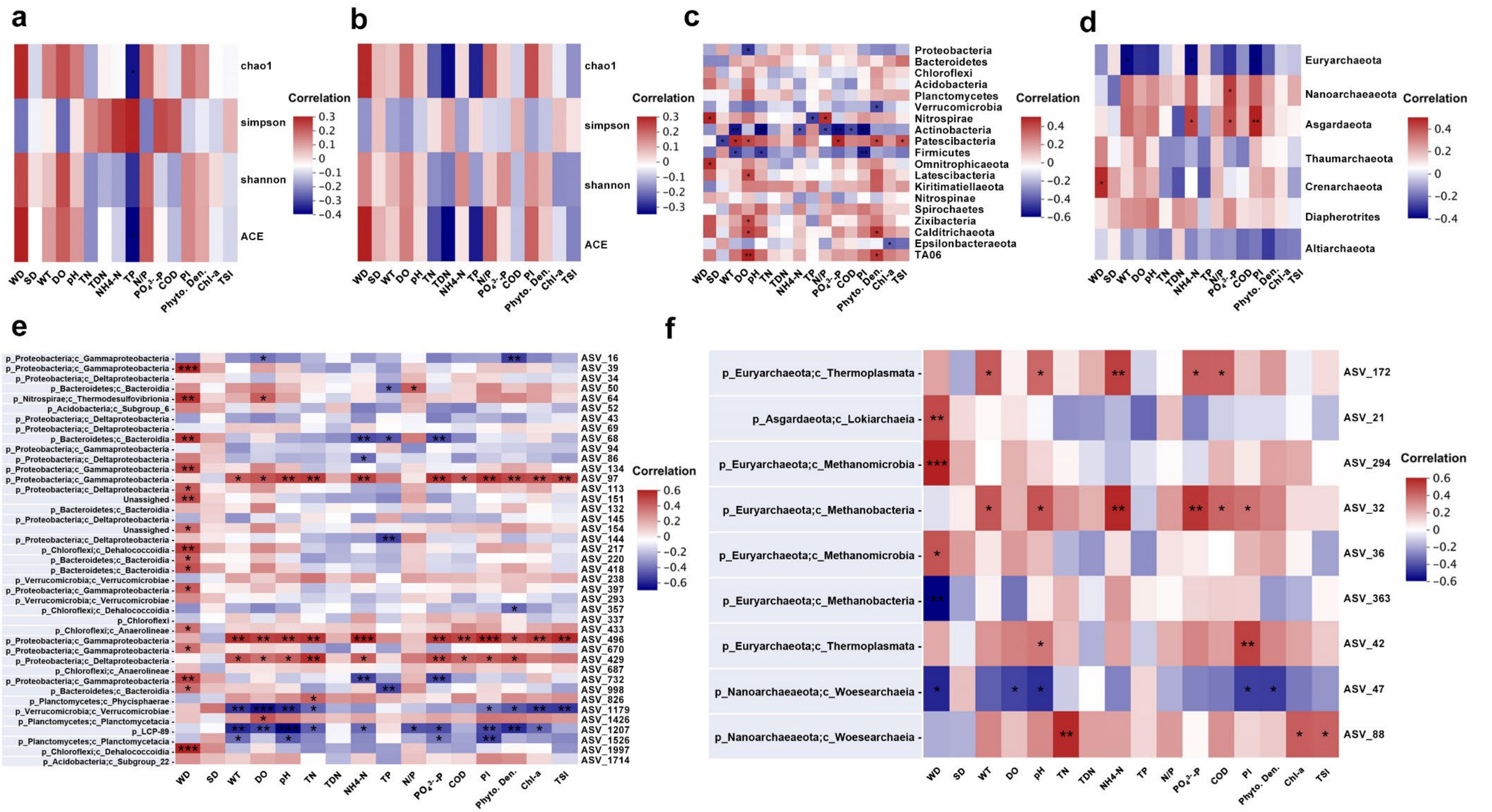

Figure S3. Heatmap showing spearman correlation between the alpha diversity index, microbial communities and environmental factors: **(a, c, e)** Bacteria; **(b, d, f)** Archaea. Blue indicates a negative correlation, while red indicates a positive correlation. ***p < 0.001, **p < 0.01, *p < 0.05.
