# Supplementary material for: Bacterial and Archaeal Communities in Erhai Lake Sediments: Abundance and Metabolic Insight into a Plateau Lake at the Edge of Eutrophication
Source: Microorganisms. 2024 Aug 8;12(8):1617. doi: 10.3390/microorganisms12081617 (PMC11356345; doi:10.3390/microorganisms12081617)
Supplement: Supplementary file 1 [file microorganisms-12-01617-s001.zip › Suporting information_Tables.pdf]

Table S1. Alpha diversity index of bacterial and archaeal communities in littoral sediments of Erhai Lake.

	Alpha diversity of bacteria						Alpha diversity of archaea					
Sample	No. of ASVs	chao1	simpson	shannon	ACE	goods_coverage	No. of ASVs	chao1	simpson	shannon	ACE	goods_coverage
HCH.1	1981	1981	0.353	2.97	2127.54	0.996	301	301	0.163	2.98	311.21	0.999
HCH.2	4419	4419	0.006	6.90	4481.16	0.998	306	306	0.138	3.22	316.06	1.000
HS.1	5449	5449	0.002	7.39	5572.05	0.996	398	398	0.098	3.53	408.22	0.999
HS.2	4394	4394	0.007	6.80	4480.66	0.997	328	328	0.135	3.17	336.50	1.000
SL.1	5581	5581	0.002	7.42	5613.69	0.999	430	430	0.087	3.65	436.89	1.000
SL.2	5350	5350	0.001	7.62	5531.91	0.995	455	455	0.094	3.77	455.00	1.000
RLY.1	3657	3657	0.011	6.44	3764.90	0.997	355	355	0.129	3.28	365.36	0.999
RLY.2	3938	3938	0.015	6.38	4021.26	0.997	352	352	0.112	3.38	368.62	0.999
SC.1	5333	5333	0.002	7.47	5394.41	0.998	424	424	0.101	3.70	435.72	0.999
SC.2	5107	5107	0.005	7.09	5107.00	1.000	475	475	0.088	3.76	487.92	0.999
JGS.1	4210	4210	0.004	6.92	4414.05	0.994	316	316	0.152	3.04	325.53	0.999
JGS.2	5276	5276	0.003	7.31	5362.72	0.997	394	394	0.099	3.54	403.33	1.000
LHQ.1	5396	5396	0.001	7.76	5565.50	0.995	368	368	0.105	3.67	376.40	1.000
LHQ.2	5058	5058	0.002	7.29	5099.96	0.998	373	373	0.105	3.41	379.58	1.000
HY.1	5859	5859	0.001	7.71	5992.81	0.996	402	402	0.091	3.69	412.02	0.999
HY.2	5001	5001	0.001	7.52	5088.66	0.997	391	391	0.123	3.36	399.96	1.000
PX.1	4927	4927	0.002	7.32	5054.51	0.996	347	347	0.148	3.14	354.64	1.000
PX.2	4405	4405	0.002	7.16	4495.05	0.997	320	320	0.142	3.11	328.97	1.000
WB.1	4581	4581	0.003	7.18	4662.28	0.997	384	384	0.135	3.22	395.98	0.999
WB.2	5566	5566	0.002	7.36	5746.05	0.995	344	344	0.153	3.12	355.94	0.999
CC.1	5247	5247	0.002	7.39	5500.34	0.993	372	372	0.098	3.44	393.06	0.999
CC.2	4362	4362	0.003	7.17	4514.17	0.995	532	532	0.060	4.02	546.68	0.999
XY.1	5098	5098	0.004	7.18	5259.77	0.995	382	382	0.125	3.38	392.97	0.999
XY.2	4642	4642	0.006	6.93	4736.02	0.997	380	380	0.138	3.24	388.09	1.000
NC.1	5707	5707	0.003	7.41	5719.91	0.999	360	360	0.107	3.49	366.16	1.000

-Continued

NC.2	5698	5698	0.001	7.64	5826.15	0.996	497	497	0.109	3.72	508.94	0.999

XH.1	5237	5237	0.002	7.33	5340.25	0.997	364	364	0.106	3.49	379.34	0.999
XH.2	5644	5644	0.002	7.58	5841.25	0.994	398	398	0.120	3.41	403.44	1.000
EBC.1	3902	3902	0.005	6.73	3986.22	0.997	271	271	0.133	3.09	281.30	1.000
EBC.2	3930	3930	0.004	6.83	4088.63	0.996	271	272	0.108	3.16	292.64	0.999
SPC.1	3950	3950	0.004	6.86	4052.79	0.997	337	337	0.115	3.29	347.75	0.999
SPC.2	4308	4308	0.004	6.98	4312.27	1.000	315	315	0.111	3.31	327.04	0.999
TS.1	4209	4209	0.002	7.35	4237.43	0.999	512	512	0.089	4.02	532.29	0.999
TS.2	4817	4817	0.005	7.00	4932.06	0.996	360	360	0.129	3.33	370.42	0.999

Table S2. Lists of keystone taxa in co-occurrence network of bacteria.

ASV ID	Zi	Pi	Types	Relative abundance	Category	Taxonomy
ASV_16	2.727	0.000	Module hubs	0.62%	Rare	p_Proteobacteria;c_Gammaproteobacteria;o_Cellvibrionales;f_Halieaceae
ASV_34	2.620	0.408	Module hubs	0.55%	Rare	p_Proteobacteria;c_Gammaproteobacteria;o_Methylococcales;f_Methylomonaceae;g_Methylobacter
ASV_39	3.804	0.500	Module hubs	0.42%	Rare	p_Proteobacteria;c_Deltaproteobacteria;o_Sva0485
ASV_43	2.773	0.000	Module hubs	0.41%	abundant	p_Bacteroidetes;c_Bacteroidia;o_Sphingobacteriales;f_Lentimicrobiaceae
ASV_50	1.075	0.676	Connectors	0.38%	abundant	p_Nitrospirae;c_Thermodesulfovibrionia;o_Magnetobacteriales;f_Magnetobacteriaceae;g_Candidatus_Magnetoovum
ASV_52	-0.641	0.641	Connectors	0.37%	Rare	p_Acidobacteria;c_Subgroup_6
ASV_64	3.930	0.180	Module hubs	0.35%	Rare	p_Proteobacteria;c_Deltaproteobacteria;o_Sva0485
ASV_68	2.970	0.000	Module hubs	0.33%	Rare	p_Proteobacteria;c_Deltaproteobacteria;o_Desulfobacterales;f_Desulfobacteraceae
ASV_69	-0.829	0.640	Connectors	0.33%	moderate	p_Bacteroidetes;c_Bacteroidia;o_Bacteroidales;f_SB-5
ASV_86	-1.174	0.639	Connectors	0.28%	Rare	p_Proteobacteria;c_Gammaproteobacteria;o_Betaproteobacteriales
ASV_94	-0.937	0.664	Connectors	0.24%	Rare	p_Proteobacteria;c_Deltaproteobacteria;o_Deltaproteobacteria_Incertae_Sedis;f_Syntrophorhabdaceae;g_Syntrophorhabdus;s_Syntrophorhabdus_aromaticivorans_UI
ASV_97	0.238	0.666	Connectors	0.23%	Rare	p_Proteobacteria;c_Gammaproteobacteria;o_Competibacterales;f_Competibacteraceae;g_Candidatus_Competibacter;s_Candidatus_Competibacter_denitrificans_Run_A_D11
ASV_113	2.023	0.664	Connectors	0.23%	Rare	p_Proteobacteria;c_Gammaproteobacteria;o_Betaproteobacteriales;f_Burkholderiaceae
ASV_132	0.486	0.647	Connectors	0.21%	Rare	p_Proteobacteria;c_Deltaproteobacteria;o_Sva0485
ASV_134	3.930	0.580	Module hubs	0.21%	Rare	Unassighed
ASV_144	5.241	0.470	Module hubs	0.20%	Rare	p_Bacteroidetes;c_Bacteroidia;o_Cytophagales;f_Microscillaceae;g_OLB12;s_Bacteroidetes_bacterium_OLB12
ASV_145	-0.700	0.720	Connectors	0.19%	Rare	p_Proteobacteria;c_Deltaproteobacteria;o_Sva0485
ASV_151	3.502	0.156	Module hubs	0.18%	Rare	Unassighed

-Continued

ASV_154	0.154	0.667	Connectors	0.17%	Rare	p_Proteobacteria;c_Deltaproteobacteria;o_Desulfarculales;f_Desulfarculaceae;g_Desulfatiglans
ASV_217	1.854	0.625	Connectors	0.17%	Rare	p_Chloroflexi;c_Dehalococcoidia;o_MSBL5
ASV_220	2.615	0.198	Module hubs	0.16%	Rare	p_Bacteroidetes;c_Bacteroidia;o_Bacteroidales;f_Bacteroidetes_vadinHA17
ASV_238	2.620	0.000	Module hubs	0.12%	Rare	p_Bacteroidetes;c_Bacteroidia;o_Bacteroidales;f_Bacteroidetes_vadinHA17
ASV_293	-0.704	0.662	Connectors	0.11%	Rare	p_Verrucomicrobia;c_Verrucomicrobiae;o_Pedosphaerales;f_Pedosphaeraceae
ASV_337	-0.238	0.727	Connectors	0.10%	Rare	p_Proteobacteria;c_Gammaproteobacteria;o_Betaproteobacteriales
ASV_357	1.808	0.625	Connectors	0.10%	Rare	p_Verrucomicrobia;c_Verrucomicrobiae;o_Pedosphaerales;f_Pedosphaeraceae
ASV_397	3.804	0.237	Module hubs	0.08%	Rare	p_Chloroflexi;c_Dehalococcoidia;o_GIF9;f_AB-539-J10
ASV_418	3.178	0.000	Module hubs	0.08%	Rare	p_Chloroflexi
ASV_429	0.715	0.667	Connectors	0.07%	Rare	p_Chloroflexi;c_Anaerolineae;o_SJA-15
ASV_433	-0.760	0.622	Connectors	0.06%	Rare	p_Proteobacteria;c_Gammaproteobacteria
ASV_496	0.000	0.663	Connectors	0.06%	Rare	p_Proteobacteria;c_Gammaproteobacteria;o_Competibacterales;f_Competibacteraceae;g_Candidatus_Competibacter;s_Candidatus_Competibacter_denitrificans_Run_A_D11
ASV_670	-0.227	0.660	Connectors	0.06%	Rare	p_Proteobacteria;c_Deltaproteobacteria;o_Sva0485
ASV_687	-1.073	0.625	Connectors	0.05%	Rare	p_Chloroflexi;c_Anaerolineae
ASV_732	0.247	0.625	Connectors	0.04%	Rare	p_Proteobacteria;c_Gammaproteobacteria;o_Methylococcales;f_Methylococcaceae;g_Methyloparacoccus;s_Methyloparacoccus_murrellii
ASV_826	-0.953	0.631	Connectors	0.03%	Rare	p_Bacteroidetes;c_Bacteroidia;o_Bacteroidales;f_SB-5
ASV_998	0.069	0.656	Connectors	0.03%	Rare	p_Planctomycetes;c_Phycisphaerae;o_CCM11a
ASV_1179	-0.995	0.656	Connectors	0.02%	Rare	p_Verrucomicrobia;c_Verrucomicrobiae;o_Pedosphaerales;f_Pedosphaeraceae
ASV_1207	-0.397	0.678	Connectors	0.02%	Rare	p_Planctomycetes;c_Planctomycetacia;o_Pirellulales;f_Pirellulaceae
ASV_1426	-1.087	0.667	Connectors	0.02%	Rare	p_LCP-89
ASV_1526	-0.715	0.640	Connectors	0.02%	Rare	p_Planctomycetes;c_Planctomycetacia;o_Pirellulales;f_Pirellulaceae;g_Thermostilla;s_Thermostilla_marina
ASV_1714	-0.516	0.621	Connectors	0.02%	Rare	p_Chloroflexi;c_Dehalococcoidia;o_Dehalococcoidales;f_Dehalococcoidaceae;g_Dehalogenimonas
ASV_1997	0.010	0.625	Connectors	0.02%	Rare	p_Acidobacteria;c_Subgroup_22

Table S3. Lists of keystone taxa in co-occurrence network of archaea.

ASV ID	Zi	Pi	Types	Relative abundance	Category	Taxonomy
ASV_36	2.860126	0.375	Module hubs	0.40%	abundant	p_Euryarchaeota;c_Thermoplasmata;o_Marine_Benthic_Group_D_and_DHVEG-1
ASV_42	2.563026	0.31405	Module hubs	0.36%	abundant	p_Asgardaeota;c_Lokiarchaeia
ASV_21	2.411544	0.625	Connectors	0.86%	abundant	p_Euryarchaeota;c_Methanomicrobia;o_Methanosarcinales;f_Methanosaetaceae;g_Methanosaeta
ASV_47	0.645033	0.625	Connectors	0.31%	abundant	p_Euryarchaeota;c_Methanobacteria;o_Methanobacteriales;f_Methanobacteriaceae;g_Methanobacterium
ASV_32	-0.9304	0.64	Connectors	0.48%	abundant	p_Euryarchaeota;c_Methanomicrobia;o_Methanomicrobiales;f_Methanoregulaceae;g_Methanolinea;s_uncultured_archaeon
ASV_88	0.253546	0.632813	Connectors	0.14%	Rare	p_Euryarchaeota;c_Methanobacteria;o_Methanobacteriales;f_Methanobacteriaceae;g_Methanobacterium
ASV_294	-0.44023	0.65625	Connectors	0.04%	Rare	p_Euryarchaeota;c_Thermoplasmata;o_Marine_Benthic_Group_D_and_DHVEG-1
ASV_363	-0.59161	0.666667	Connectors	0.03%	Rare	p_Nanoarchaeaeota;c_Woesearchaeia
ASV_172	-1.39843	0.691358	Connectors	0.07%	Rare	p_Nanoarchaeaeota;c_Woesearchaeia

Table S4. Physicochemical characterization of samples in the littoral sediments of Erhai Lake.

Samples	WD (m)	SD (m)	WT (°C)	DO (mg/L)	pH	TN (mg/L)	TDN (mg/L)	NH_4^+-N (mg/L)	TP (mg/L)	N/P	PO_4^{3-}-P (mg/L)	COD (mg/L)	PI (mg/L)	Phyto. Den. (10^4/L)	Chl-a (mg/L)	TSI
HCH.1	3.0	1.6	26.4	8.16	9.04	0.667	0.556	0.139	0.034	20	0.021	18.70	4.60	1337	0.020	47.66
HCH.2	2.5	1.0	26.8	8.58	9.02	0.657	0.567	0.139	0.035	19	0.020	18.10	4.44	1427	0.018	49.36
HS.1	5.0	1.0	26.4	8.16	9.04	0.667	0.556	0.139	0.034	20	0.021	18.70	4.60	1337	0.020	49.71
HS.2	2.8	1.0	26.8	8.58	9.02	0.657	0.567	0.139	0.035	19	0.020	18.10	4.44	1427	0.018	49.36
SL.1	3.3	1.1	26.4	8.67	9.01	0.587	0.466	0.127	0.030	20	0.015	17.00	5.32	747	0.020	48.35
SL.2	1.2	1.2	26.3	8.32	8.92	0.607	0.476	0.124	0.030	20	0.015	16.80	4.92	786	0.015	47.15
RLY.1	3.4	1.9	25.3	7.61	8.94	0.617	0.426	0.130	0.030	21	0.016	17.30	4.92	956	0.015	45.11
RLY.2	3.5	1.9	25.5	7.65	8.87	0.577	0.416	0.124	0.030	19	0.015	16.50	4.60	871	0.015	44.96
SC.1	2.1	1.4	25.6	9.11	8.97	0.582	0.506	0.116	0.036	16	0.014	17.00	4.68	2439	0.020	48.08
SC.2	3.7	1.6	26.1	9.33	9.07	0.652	0.566	0.126	0.034	19	0.014	17.20	4.56	2535	0.017	47.13
JGS.1	4.5	2.2	25.6	10.74	9.11	0.677	0.516	0.103	0.029	23	0.012	16.90	4.84	1209	0.031	47.39
JGS.2	3.6	1.5	25.7	9.87	9.12	0.637	0.466	0.112	0.027	24	0.011	16.70	4.92	1334	0.025	47.77
LHQ.1	3.4	1.7	26.1	9.33	9.07	0.652	0.566	0.126	0.034	19	0.014	17.20	4.56	2535	0.017	46.87
LHQ.2	4.7	1.6	25.6	9.11	8.97	0.582	0.506	0.116	0.036	16	0.014	17.00	4.68	2439	0.020	47.49
HY.1	4.8	1.8	26.2	8.84	9.04	0.587	0.416	0.098	0.024	24	0.011	16.50	4.52	1606	0.019	45.17
HY.2	3.5	1.8	24.5	9.48	8.83	0.567	0.456	0.103	0.027	21	0.010	16.20	4.60	724	0.021	45.91
PX.1	2.0	1.5	24.6	8.25	8.91	0.597	0.476	0.100	0.030	20	0.011	16.30	4.52	1407	0.019	46.97
PX.2	4.0	1.6	24.2	8.2	8.84	0.567	0.456	0.098	0.029	20	0.010	16.60	4.44	1073	0.023	46.97
WB.1	4.0	1.4	23.6	7.04	8.71	0.546	0.426	0.095	0.029	19	0.010	17.10	4.28	705	0.016	46.14
WB.2	3.0	1.6	23.6	7.29	8.64	0.546	0.446	0.100	0.030	18	0.010	16.60	3.96	411	0.014	45.28
CC.1	4.5	2.0	24.1	7.35	8.70	0.546	0.486	0.100	0.024	23	0.009	15.20	4.04	441	0.011	42.63
CC.2	3.0	1.7	23.8	7.62	8.61	0.536	0.436	0.104	0.026	21	0.010	15.60	4.08	450	0.012	43.73
XY.1	2.0	1.8	23.9	7.2	8.72	0.526	0.476	0.106	0.027	19	0.012	15.40	4.20	336	0.009	42.69

-Continued

XY.2	3.8	2.0	23.8	7.53	8.68	0.526	0.476	0.109	0.027	19	0.011	16.50	4.20	393	0.010	42.42
NC.1	3.2	1.4	23.9	7.2	8.72	0.526	0.476	0.106	0.027	19	0.012	15.40	4.20	336	0.009	43.79
NC.2	4.0	1.9	23.8	7.53	8.68	0.526	0.476	0.109	0.027	19	0.011	16.50	4.20	393	0.010	42.64
XH.1	3.8	1.9	23.2	7.69	8.31	0.526	0.466	0.080	0.029	18	0.012	15.20	4.04	647	0.011	43.45
XH.2	3.7	2.1	23.1	7.74	8.15	0.556	0.496	0.092	0.030	19	0.012	16.30	4.28	476	0.012	43.44
EBC.1	2.5	1.4	23.9	7.77	8.69	0.577	0.516	0.092	0.033	17	0.011	15.30	4.04	383	0.015	46.62
EBC.2	1.7	1.5	23.2	7.06	8.57	0.567	0.506	0.098	0.037	15	0.009	16.00	4.04	368	0.011	45.58
SPC.1	2.8	1.9	23.1	6.74	8.13	0.577	0.466	0.112	0.034	17	0.010	16.60	3.64	319	0.012	44.49
SPC.2	3.0	1.6	23.1	6.96	8.17	0.577	0.496	0.115	0.034	17	0.011	17.10	3.80	512	0.012	45.30
TS.1	3.5	1.8	23.3	7.37	8.50	0.546	0.446	0.100	0.037	15	0.012	16.30	3.96	401	0.013	45.21
TS.2	3.9	1.6	23.1	7.4	8.47	0.536	0.486	0.098	0.036	15	0.012	15.40	4.04	531	0.012	45.27

Abbreviation: WD: water depth; SD: water transparency; WT: water temperature; DO: dissolved oxygen; TN: total nitrogen; TDN: total dissolved nitrogen; NH_4^+-N: ammonium; TP: total phosphorus; N/P: ratio of total nitrogen to total phosphorus; PO_4^{3-}-P: orthophosphate; COD: chemical oxygen demand (COD); PI: permanganate index; Phyto. density: phytoplankton density; Chl-a: chlorophyll-a; TSI: trophic state index.
